# Low-Dosage Rumen Unprotected Creatine Precursor During the Transition Period in Single-Bearing Ewes Impacts Dynamic Changes in Muscle and Adipose Mass, Uterine Involution, and Fetal Programming Outcomes

**DOI:** 10.3390/vetsci13010097

**Published:** 2026-01-19

**Authors:** Larissa Fernandes Baia Cesar, Alfredo José Herrera Conde, Camila Muniz Cavalcanti, Bruna Vitória de Freitas Alves, Marta da Costa Sousa, Jhennyfe Nobre de Sena, Yohana Huicho Miguel, Fernando Felipe da Silva Pereira, Louhanna Pinheiro Rodrigues Teixeira, Juliana Paula Martins Alves, César Carneiro Linhares Fernandes, Aníbal Coutinho do Rêgo, Dárcio Ítalo Alves Teixeira, Davide Rondina

**Affiliations:** 1 School of Veterinary Medicine, Ceará State University (UECE), Fortaleza 60714-903, CE, Brazil; larissa.baia@aluno.uece.br (L.F.B.C.); alfredo.herrera@aluno.uece.br (A.J.H.C.); camila.muniz@uece.br (C.M.C.); bru.freitas@aluno.uece.br (B.V.d.F.A.); marta.costa@aluno.uece.br (M.d.C.S.); jhennyfe.sena@aluno.uece.br (J.N.d.S.); yohana.huicho@aluno.uece.br (Y.H.M.); fernando.felipe@aluno.uece.br (F.F.d.S.P.); juli.alves@uece.br (J.P.M.A.); darcio.teixeira@uece.br (D.Í.A.T.); 2 Experimental Biology Center, University of Fortaleza, Fortaleza 60811-905, CE, Brazil; louhannateixeira@unifor.br; 3 Centre of Health Science, University of Fortaleza, Fortaleza 60811-905, CE, Brazil; cesar.fernandes@unifor.br; 4 Animal Science Department, Federal University of Ceará, Fortaleza 60455-760, CE, Brazil; anibalcr@ufc.br

**Keywords:** creatine, fetal programming, mammary gland, ovine, pregnancy, uterine involution

## Abstract

Little is known about the reproductive impacts of the GAA, creatine precursor, in ruminants. This study aimed to evaluate the effects of GAA supply during the transition period in ewes with single pregnancies on their uterine involution. Sixteen ewes with single pregnancy were allocated to two groups: one receiving a basal diet (WGAA, *n* = 8) and another receiving a basal diet supplemented daily with 0.6 g/kg DM diet of GAA (RUGAA, *n* = 8) from 100 days of gestation to 35 days postpartum (PP). The RUGAA group showed increased feed intake after delivery and modified diet selectivity. Also, at parturition, a smaller cotyledonary surface area and lower offspring weight at birth were observed. During PP, RUGAA exhibit a slight reduction in the loin depth and backfat thickness, and higher peripheral glutathione peroxidase level. Also in PP, RUGAA expressed a greater rate of uterine lumen shrinkage, more efficient mammary parenchyma growth and a higher relative lambs growth rate. We concluded that supplementation with 0.6 g of GAA in ewes with single pregnancies improved postpartum uterine involution, but also altered the mother-fetus communication system, affecting the birth weight of the offspring. Therefore, we believe further studies are necessary to efficiently align creatine demand with the distinct physiological events that occur during gestation and the postpartum period.

## Simple Summary

Guanidinoacetic acid (GAA) is a promising nutritional supplement with recognized productive results in cattle and sheep. Thanks to its ruminal escape capacity, it represents a viable option among metabolic stimulators due to its lower cost and ease of inclusion in the diet. However, few studies have evaluated its reproductive impacts, particularly during challenging stages such as late gestation and early lactation, which encompass multiple fundamental events for both dam and offspring. Thus, in the present study, supplementation with a low dose of GAA throughout the transition period in single-bearing ewes allowed us to observe distinct effects of the product on the fetal vascular system and on the birth weight of the offspring, as well as improving the dynamics of the uterine involution process postpartum. GAA mitigated tissue depletion after parturition in females and aided in the development of the mammary gland as well as the growth of the lamb.

## 1. Introduction

In female ruminants, the end of gestation and the beginning of lactation represent critical nutritional challenges due to the simultaneous demands of multiple reproductive processes [1]. During the transition period, the main difficulty when formulating diets is the reduction in feed intake, a condition aggravated by the thermal stress common in tropical regions. Uterine growth at the end of gestation compresses the rumen and reduces its volumetric capacity, while excessive heat induces peripheral vasodilation and increased respiratory rate, raising energy expenditure and decreasing appetite [2,3]. These conditions alter hepatic and muscle metabolism, increasing the production of reactive oxygen species (ROS) and systemic inflammation. Oxidative stress compromises intestinal integrity and favors endotoxin translocation [2]. Moreover, amino acids such as arginine, methionine, and leucine are diverted from protein synthesis to antioxidant and immunomodulatory functions, reducing their availability for fetal growth and milk production [4].

In this context, targeted nutritional strategies that do not modify diet volume are promising for reproductive management. Kotsampasi et al. [5] reported that supplementation with protected methionine, choline, and betaine in Chios ewes during the periconceptional and prepartum periods improved maternal antioxidant status, increased birth weight, and enhanced reproductive efficiency without affecting dry matter intake. Ruminants utilize various gluconeogenic substrates, such as propionic acid, glycerol, lactic acid, and amino acids (alanine, glycine, arginine, and methionine), which are essential for glucose synthesis and the maintenance of energy metabolism [6].

Creatine supplementation during gestation is crucial for fetal and placental energy metabolism. Vallet et al. [7] reported that 20 g/day increased cerebral myelination in low-weight piglets, improving neonatal survival. Ellery et al. [8] observed that 5% creatine for 18 days in mice maintained metabolic homeostasis and protected against fetal injury. Sah et al. [9] demonstrated that creatine supports uteroplacental bioenergetics in sheep via the creatine kinase-phosphocreatine (Cr-CK-PCr) system, contributing to placental and fetal development and neonatal neural maturation. Studies in humans indicate that creatine is a promising neuroprotective agent for the prevention and treatment of hypoxic–ischemic encephalopathy, maintaining energy balance and reducing cerebral oxidative stress [10]. In premature infants, endogenous synthesis may be insufficient, increasing the risk of brain injury, while the high concentration of creatine in colostrum highlights a critical postnatal window. Early creatine supplementation may, therefore, promote neuroprotection and brain development in these neonates [11].

Creatine acts as an energy buffer in high-demand tissues, being essential for physical performance, metabolic health, and disease prevention [12]. Its endogenous synthesis from glycine, arginine, and methionine does not always meet physiological needs, making dietary intake necessary. The use of creatine precursors spares arginine for health and growth functions, including the production of nitric oxide [13], which acts as a vasodilator, increases blood flow to the uterus and ovaries [14], and activates pituitary nitric oxide synthase, regulating GnRH, FSH, and LH [15]. Amino acids are essential for reproductive functions such as hormone synthesis and follicular development, but their effectiveness in ruminants is limited by bacterial ruminal metabolism.

Recently, the use of GAA, a precursor of creatine, directly in feed has emerged as a promising alternative in ruminants due to its ability to escape ruminal degradation [16]. In its rumen-unprotected form, this product is more viable in terms of cost and ease of handling and application. In cattle, GAA improves productive performance, rumen fermentation, nutrient digestibility, and meat quality, and optimizes metabolic, immunological, and antioxidant responses [17,18,19], with similar effects reported in sheep [20,21,22].

Regarding its impact on reproductive function, however, information remains limited [23]. In cattle, Sousa et al. [24] observed that supplementation with 16 g/cow of GAA (0.2% of total diet DM) during the final 90 days of gestation reduced muscle mobilization and increased placental vascularization without affecting offspring performance, possibly due to reduced plasma methionine. In sheep, using the GAA dosages proposed in production studies (0.9 g/kg DM diet) for a short period before mating (10 days) improved ovarian blood supply and follicular development, but did not alter gestational outcomes [25]. The advances observed in these two recent studies suggest an effective role of GAA in reproductive responses in ruminants, but also clearly highlight the limitations of current knowledge regarding dosage and supply duration, reinforcing the need for further research.

Based on these considerations, our hypothesis is that low doses of rumen-unprotected GAA, as a creatine precursor, supplied over a prolonged period during the transition phase in ewes, may enhance nutritional signaling between mother and fetus at the end of gestation, support preparation for parturition, and assist uterine involution during the postpartum negative energy balance. Thus, the objective of the present study was to evaluate the impact of dietary supplementation with low doses of GAA during the transition period in pregnant ewes with single births on mother-fetus communication and consequent development (fetal programming), the dynamics of adipose and muscle mass mobilization in the peripartum period, uterine involution, mammary gland development before and after parturition, and the offspring’s growth potential during the suckling period.

## 2. Materials and Methods

### 2.1. Location, Animals, Pre-Experimental Conditions and Management

The study was conducted at the experimental facilities of the School of Veterinary Medicine, State University of Ceará, Brazil. All procedures were approved by the Ethics Committee on Animal Experimentation of the State University of Ceará (31032.003776/2023-32).

Forty-five adult, multiparous Santa Inês ewes from the university flock were used. All ewes were prepared for mating by synchronizing estrus and follicular waves using a hormonal protocol. Seven days before mating, an intravaginal sponge impregnated with 60 mg medroxyprogesterone acetate (Progespon^®^, Zoetis, São Paulo, Brazil) was inserted into the cranial vagina of each ewe. After six days, the sponge was removed manually, and 300 IU equine chorionic gonadotropin (eCG) (Novormon^®^, Zoetis, São Paulo, Brazil) plus 0.125 mg prostaglandin (Sincrocio^®^, Ourofino, São Paulo, Brazil) were administered intramuscularly. Twenty-four hours after prostaglandin administration and twelve hours after the onset of estrus, each ewe was mated three times at 12 h intervals using a Dorper ram of proven fertility. Pregnancy diagnosis was performed by ultrasonography 25 days after mating.

Ewes diagnosed with a single pregnancy (*n* = 16) were blocked by body weight into subgroups and kept in collective pens (two or three animals per pen) with concrete floors, receiving water and mineral salt ad libitum. All ewes received the same total mixed ration (TMR) based on corn silage and concentrate. The TMR was formulated to meet the nutritional requirements of adult single-bearing ewes [26] for each gestational phase (early and late gestation) and early lactation.

### 2.2. Experimental Design and TMR Diet Intake

**Figure 1:**
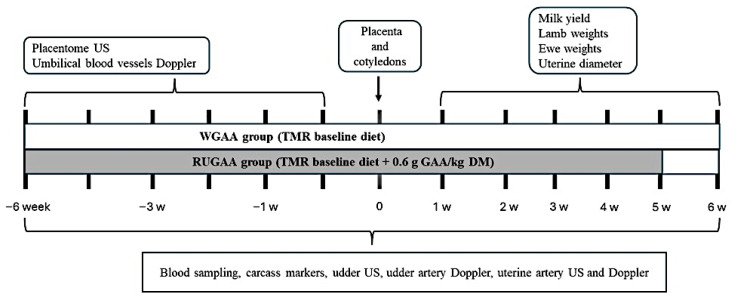
Timeline of experimental steps, including the guanidinoacetic acid (GAA) supply interval in single-bearing ewes.

On 100 days of gestation (Figure 1), ewe subgroups were assigned to two nutritional treatments with similar live weight and body condition. The overall means (±SD) for age, body weight, and body condition score were 38.3 ± 4.1 months, 49.0 ± 5.3 kg, and 2.9 ± 0.2, respectively. The control group (WGAA, *n* = 8) received the basal diet described above. The RUGAA group (*n* = 8) received the same diet supplemented daily with 0.6 g/kg dietary DM of unprotected ruminal GAA. Feed was offered twice daily at 08:00 and 15:00 until 35 days postpartum (Figure 1). Intakes were monitored daily throughout the experimental period. GAA in powder form (GuanAMINO^®^, Feed Grade 96.0%, Evonik Leading Beyond Chemistry, Hanau, Germany) was evenly distributed between the two daily meals.

The feed ingredients and average chemical composition of the diets offered in late gestation and early lactation are presented in Table 1. The particle size of the TMR was monitored using a Penn State particle separator, following the methodology described by Kononoff et al. [27]. Animal sorting patterns for the different particle sizes in each group were determined using the sorting index proposed by Leonardi & Armentano [28]. Briefly, the sorting index was calculated as the actual intake of each particle fraction expressed as a percentage of the predicted intake. Values <100% indicate selective refusals, >100% indicate preferential consumption, and =100% indicates no sorting.

### 2.3. Lambing and Lamb Weaning Management

At 145 days of gestation, lambing was induced by the intramuscular application of 10 mL dexamethasone (Azium^®^ Solução; MSD, São Paulo, Brazil) and 1 mL prostaglandin (Sincrocio^®^, Ourofino; São Paulo, Brazil). Lambs remained with their mothers during the first week to suckle colostrum. From the second week onward, they suckled in the morning and were then separated for weaning using a creep-feeding system. Lambs were weaned at 45 days of age. Feed was offered from the second postpartum week and consisted of Bermudagrass hay and a concentrate composed of 52% ground corn grain, 11% wheat bran, 32% soybean meal, 5% mineral mixture, and 5 g/kg feed-basis probiotic (Lavi Sacc^®^, Lavizoo; São Paulo, Brazil). Lambs were weighed weekly until weaning, after a 12 h fast and before morning suckling.

### 2.4. Measurements of Carcass Markers and Ewe Weight Changes Postpartum

Every two weeks until lambing and weekly thereafter (Figure 1), the depth of the lumbar region, backfat thickness, and perirenal fat thickness were measured as proposed by Morales-Martinez et al. [29] and Wang et al. [30], using a B-mode ultrasound with a 5 MHz linear probe (model Z5 Vet; Mindray Bio-Medical Electronics Co., Shenzhen, China). A 3.5 MHz convex transducer (model Z5 Vet; Mindray Bio-Medical Electronics Co., Shenzhen, China) was used for kidney imaging. Images were captured in triplicate and measured using calibrated ImageJ software (version 1.5 g, National Institutes of Health, Millersville, PA, USA). During evaluation, animals were kept stationary, areas on the right side of the body were shaved, and gel was applied as a coupling agent to improve image quality. After lambing, ewes were weighed weekly to record body mass changes during the postpartum period.

### 2.5. Ultrasonography Evaluation in Late Pregnancy

#### 2.5.1. Uterine Artery Diameter and Hemodynamic Parameters

Weekly before lambing, uterine blood flow was measured using pulsed color Doppler ultrasound (model Z5 Vet; Mindray Bio-Medical Electronics Co., Shenzhen, China) at a frequency of 5.0 MHz and color gain of 60% to visualize the uterine artery, measure its diameter, and capture pulsatile waves. Doppler velocimetric parameters were obtained from the average of three waves.

#### 2.5.2. Placentome Growth

Placentome measurements using B-mode ultrasonography were performed every seven days before lambing (Figure 1). Assessments were conducted with a 5 MHz linear transducer (model Z5 Vet; Mindray Bio-Medical Electronics Co., Shenzhen, China) and the ewe in a standing position. The average placentome diameter was calculated by randomly selecting three placentomes at each ultrasonography scan.

#### 2.5.3. Umbilical Vascular Development

Every seven days before lambing (Figure 1), the diameter of the umbilical vein and artery was measured using color Doppler ultrasonography. Assessments were performed with a 5 MHz convex transducer (model Z5 Vet; Mindray Bio-Medical Electronics Co., Shenzhen, China).

Using D-mode, vessel diameters were measured after freezing a cross-sectional image. Arteries appeared as structures of greater caliber and veins as smaller structures. For each vessel, the average of two diameters was calculated.

#### 2.5.4. Umbilical Artery Hemodynamics

Weekly, pulsatile waves from the largest umbilical artery were recorded in D-mode from an appropriate portion of the umbilical cord, and hemodynamic parameters were determined. Scanner settings were calibrated as follows: 30° angle, 3.0 MHz sampling frequency, and color gain = 32%. For each assessment, the average of three separate cardiac cycle waves was calculated.

### 2.6. Placenta and Cotyledon Traits Measured at Lambing

At lambing, the placenta was collected under sterile conditions and weighed (Figure 1). Cotyledons were classified according to their morphological characteristics, based on the degree of eversion of the hemophagous zone, and categorized as type A (cotyledon completely surrounded by maternal tissue), type B (cotyledon beginning to grow over the maternal tissue), type C (flat cotyledon with a larger hemophagous zone area), and type D (everted cotyledon), according to the classification described by Braun et al. [31], then counted and weighed by type. Five cotyledons of each type were randomly selected, weighed individually, and their length, width, and depth were measured with a caliper.

### 2.7. Postpartum Uterine Involution

After lambing, images of the gestational horn and uterine artery were collected once a week, with the first evaluation performed one day postpartum (Figure 1). During examination, ewes were kept stationary, and a B-mode ultrasound (model Z5 Vet; Mindray Bio-Medical Electronics Co., Shenzhen, China) with a 5.0 MHz linear transducer adapted to a rigid extension rod was used, following Ababneh & Degefa [32]. The probe was inserted, and the cranial bladder border was used as a reference to locate the uterus. Images were captured in triplicate and analyzed using ImageJ^®^ (version 1.54 g, National Institutes of Health, Millersville, PA, USA) to measure the largest uterine horn diameter and uterine artery diameter. For uterine blood flow evaluation, pulsed color Doppler ultrasound at 5.7 MHz was used to visualize the uterine artery and capture pulsed waves. Velocimetric parameters were obtained from the average of three waves automatically calculated by the device.

### 2.8. Mammary Gland and Artery Development

#### 2.8.1. Udder Volume

Morphometric measurements of the udder were performed using a measuring tape. Measurements were taken at three-week intervals until lambing and every three days after lambing. Depth and perimeter (cm) were recorded, and udder volume was calculated according to Haslin et al. [33].

#### 2.8.2. B-Mode Ultrasound—Mammary Gland and Pulsed Doppler—Udder Artery

Assessments of mammary gland development and hemodynamics of the mammary gland artery were performed at three-week intervals before lambing and every three days after lambing (Figure 1). Ultrasound equipment with B-mode and Doppler functions was used (model Z5 Vet; Mindray Bio-Medical Electronics Co., Shenzhen, China). Measurements were performed with animals kept in a stationary position, using a linear transducer with a frequency of 5 MHz and the B-mode function. Images were collected showing the cistern, parenchyma, and adipose pad of the mammary gland. The images were obtained following the methodology described by Haslin et al. [33], in which gel was used as a coupling agent and the transducer was applied to the external base of each teat at an approximate angle of 30° from the caudo-cranial axis, with an inclination of about 45° relative to the teat. Light and consistent pressure was applied through the transducer to minimize pressure-induced variation in the images. Using pulsed color Doppler and a convex probe with a frequency of 3.0 MHz, blood flow in the artery of each mammary gland was evaluated [34]. Initially, the arteries were visualized using the color Doppler function to capture an image for subsequent diameter measurement. Then, using the pulsed Doppler function, velocimetric parameters were collected from an average of three waves automatically calculated by the device.

### 2.9. Ewes’ Milk Yield and Lamb Growth During the Suckling Period

Milk yield was measured every three days from the fourth day postpartum until weaning, following the method proposed by Celi et al. [35], with adaptations. On the day before measurement, all lambs were separated from their mothers at 19:00. The following morning, at 07:00, each lamb was weighed before and 30 min after suckling. Milk yield was obtained from the difference between these weights, and daily production was estimated by multiplying this difference by two.

### 2.10. Blood Sampling and Biochemical Marker Analysis

Blood samples for subsequent plasma analysis were collected in the morning at three-week intervals before lambing and weekly postpartum (Figure 1). With the animal fasting, blood was drawn from the jugular vein using 4 mL vacuum tubes containing lithium heparin as an anticoagulant (FIRSTLAB^®^, Disera Tıbbi Malzeme Lojistik San. Tic. A.Ş, Izmir, Turkey). The tubes were centrifuged at 3000 rpm for 10 min, and the resulting plasma was stored at −20 °C and later analyzed for glucose, cholesterol, triglycerides, total protein, creatinine, urea, albumin, bilirubin, glutamic-oxaloacetic transaminase (GOT), glutamic-pyruvic transaminase (GPT), β-hydroxybutyrate (BHB), and glutathione peroxidase (GPx). Analyses were performed using an automated biochemical analyzer (Mindray^®^ BS 120, Mindray Biomedical Electronics Co., Shenzhen, China) and commercial kits (Bioclin^®^, Quibasa, Minas Gerais, Brazil; Randox Laboratories, Crumlin, UK), following the manufacturers’ instructions and the kits’ sensitivity for glucose, cholesterol, triglycerides, total protein, creatinine, urea, albumin, bilirubin, GOT, GPT, BHB, and GPx (1.31 mg/dL, 0.67 mg/dL, 2.58 mg/dL, 0.043 g/dL, 0.0395 mg/dL, 1.514 mg/dL, 0.0327 g/dL, 0.025 mg/dL, 1.756 U/L, 0.998 U/L, 0.100 mmol/L, and 75 U/L, respectively). Globulin concentration was calculated as the difference between total protein and albumin.

### 2.11. Statistical Analysis

Statistical analyses were performed using Statistica Software, version 13.4.0.14 (2018; TIBCO Software, Inc., Palo Alto, CA, USA). Data on dry matter intake, feed refusal, and the TMR sorting index of ewes were subjected to analysis of variance (ANOVA) using GLM procedures, with nutritional group (RUGAA, WGAA), week of the transition period (Time effect), and the group × time interaction as main effects. The ‘ewe weight’ subgroup was included as a covariate, and results were presented as corrected least square means. For parameters assessed by ultrasonography, data were analyzed using GLM procedures for repeated measures of ANOVA, with group, ultrasonography interval, and their interaction as main effects. The anatomical images (one, two, three) served as repeated measures. For placenta and cotyledon traits recorded at lambing, the GLM ANOVA included only nutritional group as an effect. Metabolite markers, udder volume, and milk yield were analyzed using GLM ANOVA with group, interval of assessment (Time effect), and their interaction as effects. Lamb weights were analyzed using GLM ANOVA with group, week, sex (male, female), and the interactions group × time and group × sex.

## 3. Results

### 3.1. TMR Diet Intake and Selective Feeding Behavior

**Figure 2:**
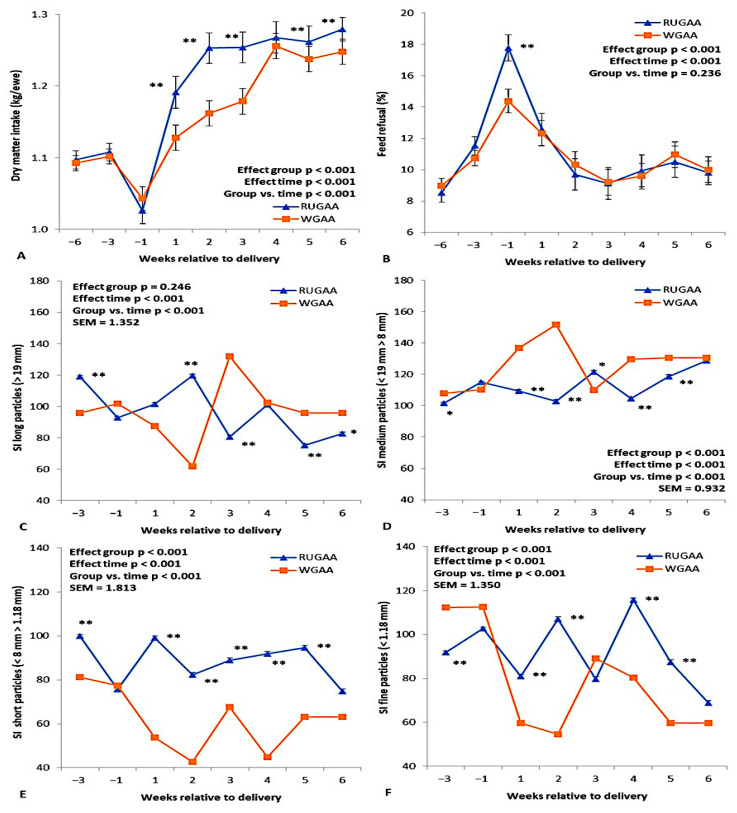
Least squares means of dry matter intake (**A**), feed refusal (**B**), and ewe variability of the sorting index (DM basis) of TMR particle size (long, (**C**); medium, (**D**); short, (**E**); and fine, (**F**)) offered during the transition period in single-bearing ewes fed a baseline TMR diet or TMR diet supplied with rumen-unprotected guanidinoacetic acid (RUGAA). Asterisks indicate where differences between groups occurred (* *p* < 0.05, ** *p* < 0.01). Time: ANOVA effect for weeks of the transition period.

Figure 2 illustrates the results for dry matter intake from the TMR diet, feed refusal, and the sorting index of TMR particle size throughout the transition period. Except for feed refusal, all parameters showed a significant interaction (*p* < 0.001) between group and experimental period. TMR dry matter intake decreased in both treatments (Figure 2A) during the last month of pregnancy and then increased until the 6th week after lambing (WAL). These increases were greater (*p* < 0.01) in the RUGAA group than in the control group, except during the 4th WAL. Along with the reduction in feed intake in the last month of pregnancy, feed refusal increased (Figure 2B) in both groups, with a more pronounced increase in the RUGAA group (*p* < 0.01).

Ewes supplemented with GAA showed a preference for long particles up to the 2nd WAL and generally lower selective refusal (*p* < 0.01) for particles with a diameter < 8 mm (Figure 2E,F). The control group showed a higher preference (*p* < 0.01) for medium particles from the first WAL (Figure 2D) and for long particles from the 3rd week WAL onwards (Figure 2C).

No differences were observed between groups for dry matter intake from the TMR expressed as a percentage of body weight (Table 2). Both groups showed positive increases during the postpartum period (Time effect, *p* < 0.001), reaching the highest value in the 6th week WAL (2.5 ± 0.03%).

### 3.2. Carcass Marker Dynamics and Ewe Body Weight Changes Postpartum

**Figure 3:**
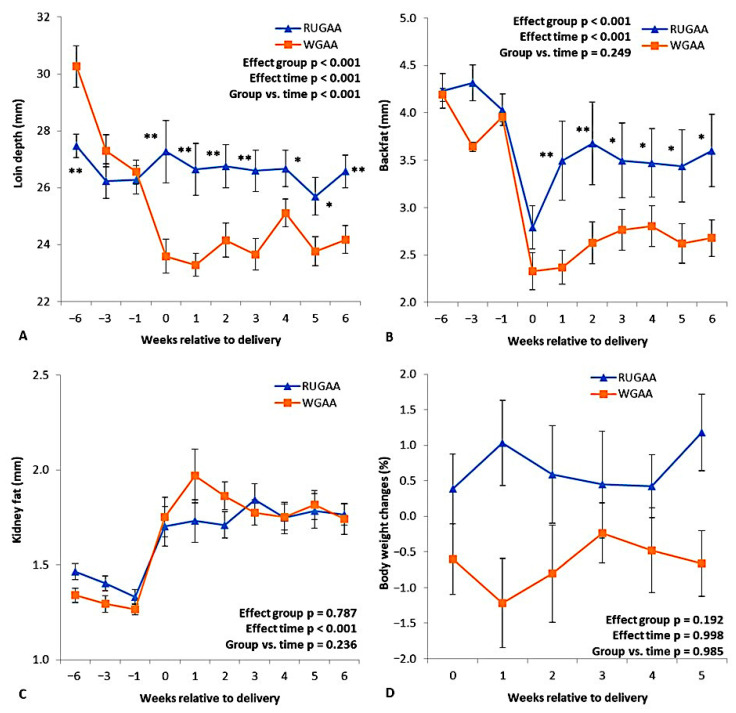
Loin depth (**A**), backfat thickness (**B**), and kidney fat (**C**) measured by ultrasonography during the transition period in single-bearing ewes fed a baseline TMR diet or TMR diet supplied with rumen-unprotected guanidinoacetic acid (RURUGAA). (**D**) shows body weight changes in ewes after lambing. Values are presented as means ± SEM. Asterisks indicate where differences between groups occurred (* *p* < 0.05, ** *p* < 0.01). Time: ANOVA effect for weeks of the transition period.

There was an interaction (*p* < 0.001) between the nutritional group and measurement intervals for lumbar area depth (Figure 3A), owing to the marked reduction (*p* < 0.01) in the thickness of this marker observed in the control group during the six weeks before lambing (WBL). The RUGAA group maintained relatively constant values throughout this period, consistently higher than those of the control. Backfat thickness showed a decline over the WBL in both groups (Figure 3B). In the RUGAA group, a recovery trend began in the first WAL and remained higher than the control up to the 6th WAL. Both groups also showed an increase in perirenal fat thickness after parturition (Figure 3C), with no difference between treatments (*p* = 0.787). No group effect was observed (*p* = 0.192) for postpartum body weight changes (Figure 3D). Despite this, the RUGAA group showed positive changes in birth weight (Figure 3D), as well as in average and total values (Table 2). Weights at lambing and weaning did not differ between the groups (Table 2).

### 3.3. Peripheral Metabolites, GPx, and BHB

**Figure 4:**
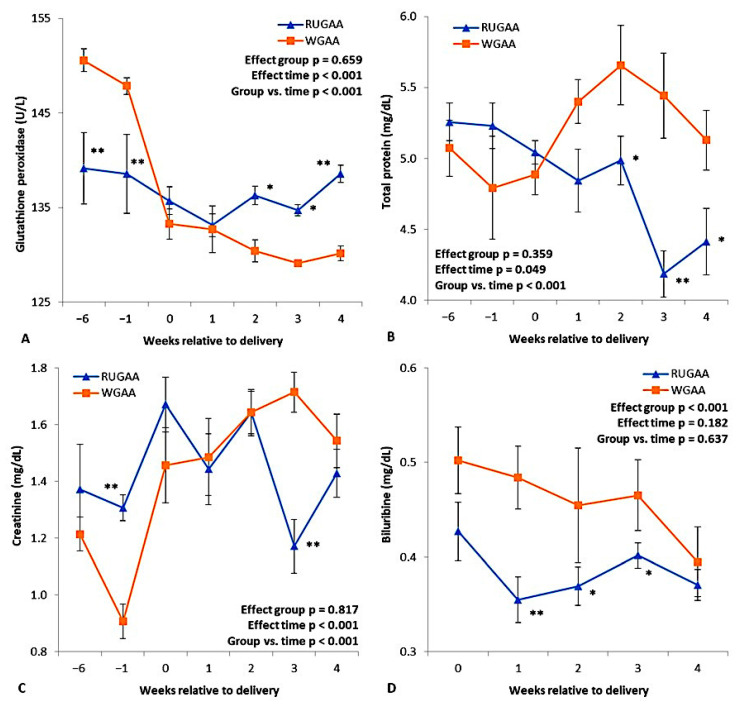
Peripheral levels of glutathione peroxidase (**A**), total protein (**B**), creatinine (**C**), and bilirubin (**D**) measured during the transition period or after lambing in single-bearing ewes fed a baseline TMR diet or TMR diet supplied with rumen-unprotected guanidinoacetic acid (RUGAA). Values are presented as means ± SEM. Asterisks indicate where differences between groups occurred (* *p* < 0.05, ** *p* < 0.01). Time: ANOVA effect for weeks of the transition period.

Figure 4 and Table 2 present the results for peripheral metabolites measured throughout the experimental period. For GPx, there was an interaction between group and sampling interval (Figure 4A; *p* < 0.001), due to a marked drop in plasma concentrations after lambing in the control group. In the RUGAA group, GPx remained higher from two to four WAL. An interaction (*p* < 0.001) was also observed for total protein (Figure 4B) and creatinine (Figure 4C), reflecting reductions in these metabolites from one to two WAL in the RUGAA group and higher creatinine in the last week before lambing. For bilirubin, supplemented animals showed consistently lower values than the control in the entire postpartum period. The RUGAA group also showed lower concentrations of glucose, albumin, and globulin (Table 2), while no differences were found for the remaining metabolites or BHB (Table 2).

### 3.4. Maternal-Fetal Communication System

#### 3.4.1. Umbilical Vascular Development and Umbilical Artery Hemodynamics

The diameter of the umbilical artery and vein, as well as total vascular surface area (Table 3), increased progressively in the WBL in both groups (Effect time, *p* < 0.001), with no differences between treatments. In contrast, peak systolic velocity of the umbilical artery (Table 3) showed an interaction between group and measurement interval (*p* < 0.001), due to a reduction at six WBL in the control group. Consequently, mean peak systolic velocity was higher in the RUGAA group than in the WGAA group. No differences were observed for end-diastolic velocity (Table 3).

#### 3.4.2. Placentome Growth, Placenta Weight, and Fetal Cotyledonary Outcome

Placentome diameter (Table 3) also showed an interaction between group and measurement interval (*p* = 0.034), due to a reduction in this parameter in the RUGAA group during the final week of pregnancy compared to the control (2.3 ± 0.04 cm vs. 2.0 ± 0.09 cm, *p* < 0.05).

No differences were found for gestation days, placental weight, placental efficiency, total cotyledon weight, or total number of cotyledons (Table 3). The RUGAA group exhibited smaller cotyledonary surface area and lower cotyledonary efficiency (*p* < 0.05, Table 3) compared with the control. Table 4 details the weights and surface areas by morphological type. The RUGAA group had lower individual weight for type C cotyledons and smaller surface areas for cotyledons B, C, and D. In this group, cotyledon surface areas did not differ among subtypes, whereas in the WGAA group, type B cotyledons had higher weight than the other subtypes.

#### 3.4.3. Uterine Artery Size and Hemodynamics

**Figure 5:**
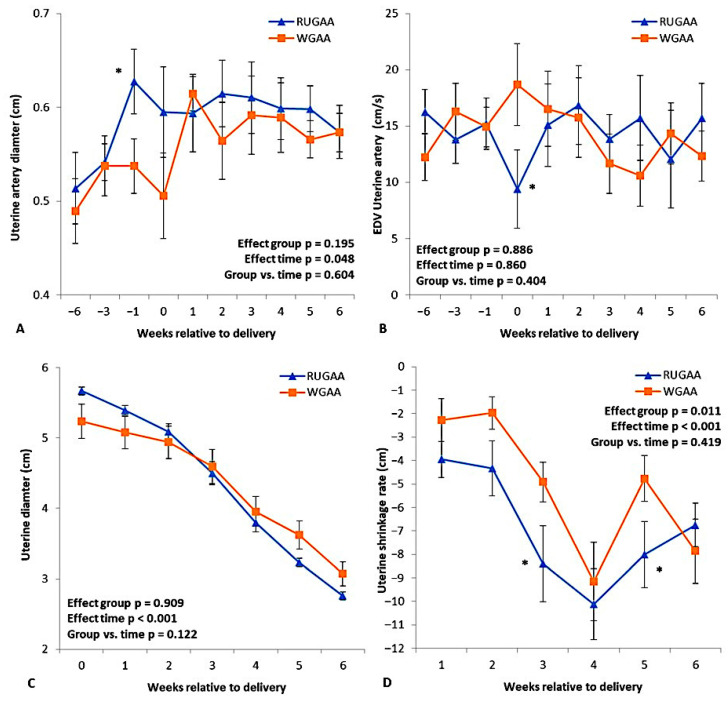
Uterine artery diameter (**A**) and end-diastolic velocity of the uterine artery (**B**) during the transition period, and uterine lumen diameter (**C**) and its shrinkage rate (**D**) recorded after lambing, measured by Doppler ultrasonography in single-bearing ewes fed a baseline TMR diet or TMR diet supplied with rumen-unprotected guanidinoacetic acid (RUGAA). Values are presented as means ± SEM. Asterisks indicate where differences between groups occurred. Time: ANOVA effect for weeks of the transition period.

The uterine artery diameter (Figure 5A) was greater in the RUGAA group during the last week of pregnancy compared with the control (*p* < 0.05), in which a contraction was observed. During the same period, end-diastolic velocity (Figure 5B) showed a transient decrease in the RUGAA group (*p* < 0.05), followed by a return to values similar to those of the WGAA group.

### 3.5. Postpartum Uterine Lumen Involution

In both groups, uterine lumen diameter (Figure 5C) decreased over the WAL (Time effect, *p* < 0.001). The shrinkage rate (Figure 5D) increased until the 4th WAL and then declined in intensity during the 5th and 6th WAL. The RUGAA group showed a higher shrinkage rate (+25%) than the WGAA group (−6.9 ± 0.54 mm/day vs. −5.2 ± 0.51 mm/day; *p* = 0.011).

### 3.6. Mammary Gland Development, Udder Volume, and Milk Yield Outcomes Postpartum

**Figure 6:**
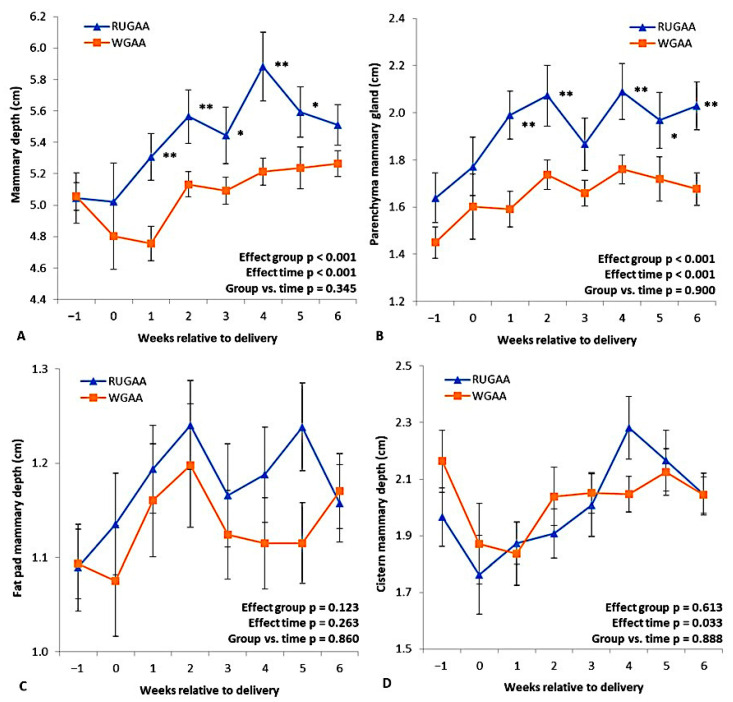
Mammary gland depth (**A**), parenchyma thickness (**B**), fat pad thickness (**C**), and cistern depth (**D**) measured by ultrasonography during the transition period in single-bearing ewes fed a baseline TMR diet or TMR diet supplied with rumen-unprotected guanidinoacetic acid (RUGAA). Values are presented as means ± SEM. Asterisks indicate where differences between groups occurred (* *p* < 0.05, ** *p* < 0.01). Time: ANOVA effect for weeks of the transition period.

Figure 6 illustrates the ultrasonographic measurements of the mammary gland (MG). The RUGAA group had greater MG depth from one to five WAD (Figure 6A). Fat pad depth (Figure 6C) and cistern depth (Figure 6D) did not differ between groups, while parenchymal thickness was greater in the RUGAA group from one to six WAL (Figure 6B). The MG artery (Table 5) had a smaller diameter in the RUGAA group (*p* = 0.019), and interactions were observed for peak systolic velocity (*p* < 0.001) and end-diastolic velocity (*p* = 0.048), reflecting lower values in the RUGAA group between six and three WBL.

Table 5 presents the results for udder volume and daily milk yield postpartum. Both variables increased over the WAL (Time effect, *p* < 0.001); however, RUGAA had lower milk yield than WGAA (*p* = 0.002).

### 3.7. Lamb Growth Outcome During the Suckling Period

The RUGAA group showed lower birth weight (Table 6) than the control (*p* = 0.043). However, weights at weaning and daily weight gain did not differ between treatments. Both groups exhibited positive weight gain during the suckling period (Effect time, *p* < 0.001), and RUGAA lambs showed higher relative growth compared with weight at birth (*p* = 0.039).

## 4. Discussion

The results showed that the use of GAA at low dosages, but for longer periods, as applied during the transition phase, consistently influences the reproductive response of pregnant ewes. This period is marked by several events essential to fetal and maternal health, as well as to the ability of females to meet postpartum nutritional demands. In this context, the findings indicate that GAA at the proposed dosage induces a combination of effects that must be interpreted carefully.

GAA effectively modulates feeding behavior by encouraging TMR consumption through a greater preference for smaller particles. During the final third of gestation, fetal growth reduces rumen capacity, limiting dry matter intake [36]. Under such conditions, smaller TMR particles become advantageous because they have a higher nutrient density, especially non-fibrous carbohydrates and protein, and provide a larger surface area for ruminal fermentation [36,37]. Turkestani et al. [36] reported that sheep fed corn silage with smaller particles exhibited greater dry matter intake, higher ruminal ammonia concentration, increased microbial protein synthesis, and enhanced protein digestibility. These responses were attributed to rapid ruminal degradation and increased production of volatile fatty acids, particularly propionate, which is fundamental to meeting lactation energy requirements. Mendonça et al. [37] also highlighted that corn silages with reduced particle size and inoculated with lactic bacteria have greater digestibility and aerobic stability, contributing to improved nutritional use by ruminants during the transition period. Leonardi & Armentano [28] state that ruminants tend to select finer TMR particles, which concentrate the most fermentable and energetic ingredients, such as grains and bran.

Supplementation with GAA appears to intensify this process by stimulating creatine biosynthesis and supporting the phosphocreatine system, which serves as a reserve of high-energy phosphates for ATP regeneration. Creatine synthesis depends on arginine, glycine, and methionine, is mediated by the AGAT and GAMT enzymes, and is regulated by energy demand and precursor availability [38]. Zhang et al. [21] reported that lambs supplemented with GAA have higher concentrations of creatine, IGF-I, and insulin, greater nutrient flow to the duodenum, and improved growth performance. In cattle, GAA stimulates the production of volatile fatty acids, especially propionate and butyrate, and improves the meat lipid profile [17].

Creatine acts as an intracellular energy buffer with antioxidant properties, essential for maintaining metabolic homeostasis during physiological stress, such as postpartum [12]. Thus, GAA supplementation suggests an improvement in energy metabolism and a modulation of ingestive behavior that encourages the selection of denser and more fermentable particles to meet increased energy requirements, particularly during critical periods such as the end of gestation and the beginning of lactation.

In the present study, increased feed intake was reflected in the metabolic profile and in the dynamics of adipose and muscle tissue throughout the experimental period. In the GAA group, substantial muscle depletion was not observed, in contrast with the control group during the transition from gestation to postpartum. This mechanism was supported by the reduction in plasma protein fractions and glucose. These adjustments likely contributed to maintaining higher circulating levels of glutathione peroxidase, as observed in the results. Likewise, in the GAA group, the reduction in lumbar subcutaneous fat postpartum was smaller, resulting in a more moderate body weight change. Postpartum, ruminants experience a marked negative energy balance, which induces an adaptive response prioritizing the mobilization of subcutaneous and visceral fat as the primary energy source. This strategy is well recognized for its high energy density and lower functional cost, being essential to sustain milk production and preserve metabolic homeostasis [39,40].

Adipose tissue lipolysis is intensified by catabolic hormones such as cortisol and catecholamines, promoting the release of free fatty acids (FFAs) into circulation. These FFAs are taken up by the liver and metabolized to generate energy and ketone bodies, such as BHB, which become particularly important during hypoglycemia. Feng et al. [41] demonstrated that, in addition to acting as an energy substrate, BHB also modulates hepatic gluconeogenic enzymes, including FBP1 and PCK1, through β-hydroxybutyration, supporting glucose regulation during BEN. When lipolysis alone is insufficient to meet metabolic demands, the body resorts to skeletal muscle mass as a secondary source of amino acids such as alanine and glutamine for hepatic gluconeogenesis, helping maintain glycemia and sustain milk protein synthesis [40,42].

Dietary supplementation with GAA has been associated with increased endogenous creatine synthesis, which plays a central role in maintaining cellular energy balance through ATP regeneration via the phosphocreatine system. Recent studies demonstrate that GAA supply stimulates the activity of the AGAT enzyme in the liver, increasing plasma levels of creatine and its precursors (arginine, citrulline, and ornithine), as observed by Sousa et al. [24] in beef cows in the final third of gestation. This additional creatine is primarily directed to tissues with high energy demands, such as skeletal muscle, where it acts as a reserve of high-energy phosphates, reducing the need for protein degradation and optimizing the mobilization of amino acids for productive functions, such as lactation and uterine recovery. GAA improves muscle metabolism and reduces ribeye area variation (REA), suggesting less muscle catabolism. Creatine also influences cell signaling via mTOR and Akt, promoting protein synthesis and preserving muscle mass. By reducing the need for excessive mobilization of muscle and adipose tissue, GAA contributes to a more efficient metabolic adaptation during negative energy balance, favoring productive performance without compromising the integrity of body tissues [17,19].

It is known that the intense mobilization of body tissues and the activation of inflammatory pathways occur due to negative energy balance, establishing a state of oxidative stress in ruminant animals during the postpartum period. This scenario increases the production of reactive oxygen species (ROS), requiring an efficient antioxidant response to preserve cellular integrity and liver function. Glutathione peroxidase (GPx) is one of the main antioxidant enzymes involved in this process, acting in the reduction in lipid and hydrogen peroxides through the oxidation of reduced glutathione (GSH) [43].

Several studies demonstrate that GPx levels vary substantially during the postpartum period. Mikulková et al. [44] observed that dairy cows showed a significant reduction in GPx activity at 14 days postpartum, accompanied by a drop in GSH levels and an increase in oxidized glutathione (GSSG), indicating impaired hepatic antioxidant capacity. Similarly, Pisťková et al. [45] reported a decrease in GPx activity between 10 and 15 days postpartum, associated with a reduction in antioxidant vitamins such as A, E, and β-carotene. In sheep, Dunière et al. [46] found that GPx activity increased postpartum as an adaptive response to oxidative stress, being positively modulated by supplementation with live yeast. This intervention reduced malondialdehyde (MDA) levels and improved total antioxidant status, suggesting that GPx is activated as a compensatory mechanism. These findings reinforce the importance of GPx as a sensitive marker of oxidative stress in ruminants during the transition period.

During the gestation period, the highest level of peripheral creatinine in the RUGAA group occurred in parallel with an increase in uterine vascular diameter and uterine arterial hypotension, verified by the drop in the end-diastolic peak velocity. During the same period, an alteration in the umbilical artery systolic peak was also observed, as well as in the hemodynamic parameters of the mammary gland artery. These events were probably the cause of the reduction in the surface area of the placentome, type B, C and D cotyledons, and the cotyledonary vascular capillary system, which in turn may have contributed to the lower live weight at birth recorded in the offspring.

These maternal and fetal hemodynamic alterations are particularly relevant, as the development and expansion of placental vascularization depend directly on efficient uterine blood flow throughout gestation, especially during the final third. Placental vascular growth is essential for the substantial increases in placental blood flow during gestation, and the pattern of vascular development can differ between the maternal (caruncle) and fetal (cotyledon) compartments. In ewes during late gestation, the cotyledon exhibits marked increases in the area, number, and surface density of capillaries, although capillary size decreases compared to the caruncle [47]. By the end of gestation, approximately half of the increased cardiac output is directed to the gravid uterus and mammary gland, with the uterine artery throughout gestation being responsible for transporting oxygen and nutrients from the maternal circulation to the placental bed, a condition essential for adequate fetal growth [48]. Efficient uterine blood flow maintains a healthy intrauterine environment and preserves placental functionality. This occurs through two primary mechanisms: maternal blood supplies nutrients and removes metabolites from the fetus and placenta; and the flow rate in the uterine artery determines the amount of oxygen available at the maternal-fetal interface, directly influencing placental health and fetal development [49]. Adequate angiogenesis and expansion of placental vascularization are crucial for increasing the exchange surface area and allowing the maternal system to support accelerated fetal growth in the final third of gestation [50].

In Doppler evaluations of arteries, a relative reduction in peak systolic pressure in the uterine artery indicates increased uteroplacental resistance and reduced effective blood flow to the placental bed [51]. In sheep, restricting uterine perfusion in the final third of gestation results in reduced placental mass, decreased umbilical blood flow, and consequent intrauterine growth restriction [52]. This process decreases the expression and production of angiogenic and placental growth factors, including insulin-like growth factor (IGF) and its binding proteins (IGFBPs), which can impair vascular expansion in the placenta and limit the development of the capillary network [53]. IGF stimulates the expression of angiogenic factors, particularly Vascular Endothelial Growth Factor (VEGF) and its receptor KDR/VEGFR2, and modulates angiopoietin activity, promoting endothelial proliferation and vascular remodeling in placentomes. This enhances capillarity and maternal-fetal perfusion [53].

Furthermore, regarding the results found in the types of cotyledons, Erichsen et al. [54], describes the physiological relevance of each placentome type differs and can be observed through variations in gene expression. Type B placentomes show higher expression of GLUT1, SN1, and SLC6A14, indicating a greater capacity for glucose and amino acid transport. Type C placentomes exhibit higher expression of VEGFR2 and HIF1α, suggesting greater stimulation of angiogenesis and arteriogenesis and a more efficient response to hypoxia. Additionally, type C and D placentomes display a larger area of maternal-fetal interdigitation and increased vascularization, which increases the efficiency of nutrient transfer [55] and supports compensation for adverse maternal conditions, as greater placental vascularization is associated with improved nutrient transport [56]. In cattle, Vonnahme et al. [50] reported that maternal undernutrition reduces fetal weight and that undernourished females have a higher proportion of type C and D placentomes. The authors suggest that these morphological types may have a compensatory role, as they are potentially more functional and more vascularized than type A and B placentomes, favoring maternal-fetal exchange and mitigating the negative effects of nutritional restriction on fetal growth.

The use of GAA during gestation in sheep has not been investigated, and information regarding appropriate dosages, administration length, and effects during critical periods such as the peripartum remains unavailable. In rats, GAA consumption increased plasma creatine and markedly elevated plasma homocysteine [57]. In ruminants, studies have also shown that GAA can increase homocysteine concentrations. Ardalan et al. [58], working with Holstein steers receiving abomasal infusions of 7.5 or 15 g/day of GAA for 10 days, observed that GAA increased plasma creatinine and homocysteine, whereas methionine supplementation reduced circulating homocysteine. In the study by Sousa et al. [24], supplementation with 16 g/cow of GAA (0.2% of total diet DM) during the final 90 days of gestation in cattle increased nitric oxide and placental vascularization without affecting creatinine concentrations; however, it increased serum homocysteine and induced methionine deficiency.

Creatine synthesis from GAA occurs mainly in the liver through a reaction catalyzed by GAMT. In this process, GAA receives a methyl group from S-adenosylmethionine, which is derived from methionine, resulting in the production of creatine and adenohomocysteine. Creatine is then transported from the liver to tissues with high energy demands, such as skeletal muscle, brain, and reproductive organs, to support intracellular ATP turnover and maintain an adequate ATP/ADP ratio. S-adenosylmethionine can be hydrolyzed to form homocysteine and adenosine, and homocysteine may be metabolized to cysteine, remethylated to methionine, or exported to the circulation [24,57]. Obtaining creatine from GAA consumes more methyl groups than all other methylation reactions combined, which can lead to methyl group deficiency [57]. With sufficient availability of methyl groups, homocysteine is remethylated to methionine-by-methionine synthase or betaine-homocysteine methyltransferase. However, under conditions of methyl group deficiency, homocysteine may not be converted to methionine, resulting in increased plasma homocysteine [24,58].

Sousa et al. [24] note that GAA methylation is not a regulated process and that creatine synthesis proceeds according to the amount of GAA available. In this context, the dosages currently used in sheep during challenging physiological phases such as the transition period may still be excessive, potentially depleting substantial quantities of methyl groups and, under certain conditions, inducing methyl group deficiency.

In the present study, hypotension in the uterine artery and a reduction in the surface area of the placentome and cotyledons were observed. Regarding these effects, Arutjunyan et al. [59] reported that increased blood homocysteine concentrations in Wistar rats induced oxidative stress in the placenta and altered pro-angiogenic and growth factors (VEGF-A, MMP-2, VEGF-B, BDNF, and NGF), which may contribute to placental growth retardation. Conversely, nitric oxide (NO) is an essential signaling molecule synthesized by nitric oxide synthase (NOS) and functions as the principal endothelial vasodilator. In addition to regulating vascular tone, NO protects blood vessels by inhibiting endothelial activation, macrophage infiltration, foam cell formation, platelet aggregation, inflammation, and vascular wall remodeling, thereby preventing endothelial injury and dysfunction [60]. Elevated homocysteine impairs endothelial NO synthesis by inhibiting the enzyme dimethylarginine dimethylaminohydrolase (DDAH), resulting in the accumulation of asymmetric dimethylarginine (ADMA), a potent NOS inhibitor. Homocysteine can also activate protein kinase C, directly reducing NOS activity. Diminished NO bioavailability promotes endothelial injury, increasing oxidative stress and vascular inflammation [61]. As a result, blood flow to irrigated tissues decreases, compromising perfusion and vascular homeostasis.

Despite the findings related to fetal vascularization, from a reproductive standpoint, the GAA group showed a more favorable uterine involution dynamic, likely driven by improved energy balance and, consequently, greater nutrient flow. Uterine hypotension before lambing may have also contributed, considering that uterine blood flow is one of the major determinants of the involution process. The time required to complete uterine involution (between the fourth and fifth week postpartum) depends on factors such as parity, lactation status, nutrition, breed, and season [62,63]. According to Elmetwally & Bollwein [64], uterine blood flow in sheep decreases sharply in the postpartum period, particularly during the first nine days after lambing. On the 3rd day postpartum, the authors described a reduction of approximately 80% in blood flow volume, illustrating the rapid hemodynamic change that accompanies involution. This marked drop is associated with a substantial reduction in uterine size and weight, increased vascular resistance, and the mechanical effect of myometrial contractions, which compress uterine vessels. The decline in blood flow also reflects the reduction in uterine volume by more than 50% and the elimination of most lochia during the first postpartum week.

Myometrial contractions and lochia elimination immediately after lambing are of paramount importance for uterine involution [65], and these contractions depend on the interaction between ATP and its purinergic P2 receptors (P2X7Rs, P2X1) [61]. According to Zafrah & Alotaibi [61], elevated ATP levels strongly potentiate myometrial contractions. Studies in sheep have shown that dietary GAA increases circulating creatine concentrations [21,66,67]. According to Liu et al. [68], following the addition of GAA to the diet, GAA receives a methyl group from S-adenosylmethionine and is converted to creatine. Creatine and phosphocreatine mediate the transfer of high-energy phosphates between ADP and ATP in tissues with high energy demands, increasing energy availability in lactating cows. Zhang et al. [69] also reported that dietary GA increased ATP concentrations in lamb muscle.

The inclusion of GAA also contributed to the dynamics of mammary gland growth in the postpartum period. The increase in mammary gland size was driven by substantial expansion of parenchymal tissue; however, this effect did not translate into greater milk production in the present study, which affected lamb performance during suckling. Despite this, lambs in the GAA group exhibited superior relative growth, suggesting a possible compensatory effect of milk components in this group.

Cell proliferation in the mammary gland is controlled by growth factors such as IGF-I, which promote tissue growth [70,71]. The exponential increase in mammary gland volume with advancing gestation reflects the intense expansion of the epithelial compartment, and around 90 days of gestation, alveolar cells begin synthesizing lipid and protein components of milk, indicating that secretory differentiation is already underway [70]. On the other hand, milk production by mammary epithelial cells is a process highly dependent on ATP, whose availability results from the expansion and activation of mitochondrial biogenesis throughout gestation and lactation. Studies in mammals have shown that the mammary gland dynamically adjusts its bioenergetic function during these periods. In mice. According to Hadsell et al. [72], studies in rats and cows observed variations in ATP concentrations throughout lactation, reinforcing that mitochondrial function follows the metabolic demands of the gland [72]. The relevance of ATP becomes even more evident due to its direct participation in the biosynthesis of milk components and in the active transport of ions and solutes, processes intensified in the peripartum period. During this period, the increased activity of ATPases, such as Ca^2+^-ATPases, indicates that energy demand grows in parallel with the activation of the secretory apparatus, and that mitochondrial and enzymatic adaptations constitute an essential bioenergetic reprogramming to prepare the mammary gland for the high metabolic demand of milk production [73]. In this context, as described earlier, supplementation with GAA increases the bioavailability of creatine, which, consequently, can raise cellular ATP levels and enhance energy availability for physiological processes dependent on this molecule.

The growth of the mammary gland before parturition occurs along with angiogenesis and vascular remodeling, mediated by vascular endothelial growth factors (VEGF), as observed in the mammary parenchyma of mice [71,74,75].

According to Barbagianni et al. [76], during the last month of gestation, the mammary gland of sheep undergoes marked vascular development parallel to parenchymal growth, evidenced by the increase in the diameter of the external pudendal artery and the expansion of intramammary vessels. This process is regulated by hormones that stimulate late mammary tissue growth, particularly estrogens and progesterone, whose action is reinforced by the progressive reduction in vasoconstrictor hormones at the end of gestation [77]. Consistently, Doppler ultrasound parameters demonstrate a continuous increase in blood perfusion as lactogenesis approaches, reflecting a greater blood volume and a greater number of erythrocytes in motion. This increase is essential because blood provides the necessary precursors for the synthesis of milk components; thus, blood flow increases progressively as the gland prepares for milk secretion. On the other hand, reductions in blood flow can compromise alveolar oxygenation, decreasing oxygen transfer and contributing to lower milk production in ewes subjected to reduced mammary perfusion [76].

Consistent evidence demonstrates that mammary parenchyma volume is a central determinant of productive capacity in ruminants. Collier & Bauman [78] highlight that, in ruminants, the potential for milk production is strongly conditioned by the amount of secretory tissue present in the gland, reflecting the number of functional epithelial cells, and by the metabolic activity of these compartments. Thus, greater parenchymal mass implies a greater capacity for milk synthesis and secretion. Conversely, milk composition depends much more on genetic, nutritional, and physiological factors than on gland size.

## 5. Conclusions

The inclusion of 0.6 g/kg DM of GAA in the diet during the transition period of single-bearing sheep stimulated feed consumption and particle selectivity, which allowed for reduced adipose and muscle mass losses postpartum and better uterine involution dynamics. Despite these results, the availability of creatine at the end of gestation in this study corresponded to a uterine and umbilical vascular alteration that resulted in reduced cotyledonary development and lower offspring weight at birth. The low dosage of GAA used allowed for better mammary gland tissue development, but in this specific case, it did not translate into increased milk production. Taken together, the evidence suggests the use of GAA in postpartum reproductive recovery in sheep, but also highlights the need for further investigations using different dosages and periods during gestation and postpartum in order to align the multiple productive and reproductive events of the animal.

## Figures and Tables

**Table 1:** Proportion of ingredients and chemical composition of the TMR basal diet (g/kg of DM) offered during transition period in single pregnant ewes fed supply with ruminal unprotected guanidinoacetic acid (RUGAA), or fed with baseline TMR diet (WGAA).

Attributes	TMR Diet	
	Late Pregnancy	Early Lactation
Ingredient, g/kg of DM		
Corn silage	700	700
Ground corn grain	140	60
Soybean meal	40	140
Wheat bran	100	90
Mineral mixture *	20	20
Chemical fraction		
Dry matter, g/kg as-fed basis	551.6	553.3
Crude protein, g/kg of DM	98.4	133.3
Ether extract, g/kg of DM	33.0	31.3
Ash, g/kg of DM	59.1	64.8
Neutral-detergent fiber, g/kg of DM	493.7	491.8
Acid-detergent fiber, g/kg of DM	233.9	236.1
Non-fibrous carbohydrates, g/kg of DM	333.1	429.4

* Ovine Premix. Premix containing (per kg): Ca 350 g, P 35 g, K 12.5 g, Na 20 g, Mg 10 g, Zn 850 mg, Fe 1100 mg, Co 15 mg, S 10 mg, F 244 mg, I 20 mg, Mn 700 mg, Se 7 mg, Vitamin A 60,000 IU, Vitamin D 15,000 IU, Vitamin E 450 IU.

**Table 2:** TMR dry matter intake, body weight changes and peripheral metabolites pattern recorded during transition period, in single pregnant ewes fed supply with ruminal unprotected guanidinoacetic acid (RUGAA), or fed with baseline TMR diet (WGAA).

Attributes	Group			*p*-Value		
	WGAA	RUGAA	SEM	Group	Time	G vs. T
Dry matter intake, %/BW *	2.3	2.4	0.011	0.325	<0.001	0.121
Ewes body weight changes						
Delivery, kg	48.2	49.9	2.484	0.747	-	-
Weaning, kg	47.9	50.3	2.401	0.632	-	-
Average weight changes, kg	−0.3	0.4	0.539	0.537	-	-
Total weight change from delivery, %	−0.7	1.2	1.192	0.461	-	-
Energy metabolism						
Glucose, mg/dL	63.7	59.8	1.236	0.032	<0.001	0.222
Cholesterol, mg/dL	53.2	53.7	1.220	0.931	<0.001	0.839
Triglycerides, mg/dL	18.4	17.3	0.781	0.221	<0.001	0.316
BHB, mmol/L	0.34	0.34	0.011	0.864	0.872	0.763
Proteins						
Albumin, mg/dL	2.6	2.3	0.049	0.002	0.133	0.426
Globulin, mg/dL	2.7	2.4	0.090	0.039	0.024	0.047
Kidney injury						
Urea, mg/dL	24.8	27.3	0.999	0.068	<0.001	0.824
Liver injury						
Albumin/globulin ratio	1.0	1.1	0.048	0.759	0.059	0.134
GOT, U/L	76.6	81.2	1.877	0.503	0.001	0.110
GPT, U/L	16.3	16.0	0.455	0.535	0.283	0.404

* BW: body weight values as shown least squares means; BHB: b-hydroxybutyrate GOT: glutamic-oxaloacetic acid transaminase; GPT, glutamic-pyruvic acid transaminase; Time: ANOVA effect for weeks of transition period intervals.

**Table 3:** Umbilical blood vessels system, hemodynamic Doppler parameters of umbilical artery and placentome size, measured by ultrasonography during late pregnancy. Placental and cotyledon traits recorded at delivery in single pregnant ewes fed supply with ruminal unprotected guanidinoacetic acid (RUGAA), or fed with baseline TMR diet (WGAA).

Attributes	Group			*p*-Value		
	WGAA	RUGAA	SEM	Group	Time	G vs. T
Umbilical vascular development						
Artery diameter, cm	7.2	7.4	0.164	0.351	<0.001	0.425
Vein diameter, cm	5.6	5.9	0.142	0.157	<0.001	0.631
Total vascular area, cm^2^	135.1	145.3	6.297	0.211	<0.001	0.435
Umbilical artery hemodynamic						
Peak systolic velocity, cm/s	48.0	50.3	0.540	0.004	<0.001	0.001
End-diastolic velocity, cm/s	24.1	22.9	1.089	0.505	0.720	0.463
Placentome						
Placentome diameter, cm	2.1	2.2	0.030	0.413	0.868	0.034
Reproductive features at delivery						
Day of pregnancy, days	147	146	0.241	0.335	-	-
Placenta weight, g	485.7	542.9	47.29	0.646	-	-
Placental efficiency ratio *	9.6	7.9	0.699	0.248	-	-
Total cotyledon weight, g	179.0	146.5	14.75	0.175	-	-
No of cotyledons, *n*	72.1	61.1	4.283	0.175	-	-
Total cotyledon surface area, mm^2^	7589.2	4941.3	536.6	0.008	-	-
Cotyledon efficiency ratio **	593.0	843.8	58.65	0.024	-	-

* Lamb weight/placental weight ratio; ** lamb weight/cotyledons surface area ratio; Time: ANOVA effect for weeks of pregnancy period intervals.

**Table 4:** Weight and surface area of fetal cotyledon subtypes recorded at delivery in single pregnant ewes fed supply with ruminal unprotected guanidinoacetic acid (RUGAA), or fed with baseline TMR diet (WGAA).

Attributes	Cotyledon Subtype				
	A	B	C	D	SEM
Average cotyledon weight, g					
WGAA	1.9 a	2.8 b	3.7 cA	2.4 d	0.109
RUGAA	1.9 a	2.6 ab	3.1 bB	2.0 a	0.134
Cotyledon surface area, mm^2^					
WGAA	89.7 a	125.0 bA	92.1 aA	96.5 aA	3.190
RUGAA	80.3	87.9 B	76.1 B	78.9 B	2.572

a, b, c, d: Columns with different letters between subtypes for each group, differ (*p* < 0.05); A, B: rows with different letters between groups for each subtype, differ (*p* < 0.05).

**Table 5:** Mammary gland artery size and hemodynamics parameters recorded during transition period, udder volume and milk traits yield estimated after delivery in single pregnant ewes fed supply with ruminal unprotected guanidinoacetic acid (RUGAA), or fed with baseline TMR diet (WGAA).

Attributes	Group			*p*-Value		
	WGAA	RUGAA	SEM	Group	Time	G vs. T
Mammary gland artery						
Diameter, mm	57.5	55.9	0.594	0.019	<0.001	0.306
Peak systolic velocity, cm/s	40.7	41.2	0.351	0.444	<0.001	<0.001
End-diastolic velocity, cm/s	20.1	20.1	0.348	0.595	<0.001	0.048
Udder trait and milk yield						
Udder volume, cm^3^	3716.9	3537.3	100.6	0.134	<0.001	0.993
Milk yield, kg/day	1.2	1.1	0.022	0.002	<0.001	0.799

Time: ANOVA effect for weeks of period intervals.

**Table 6:** Growth outcome in lambs delivered from pregnant ewes fed supply with ruminal unprotected guanidinoacetic acid (RUGAA), or fed with baseline TMR diet (WGAA).

Attributes	Group			*p*-Value				
	WGAA	RUGAA	SEM	Group	Time	Sex	G vs. T	G vs. S
Body weight at lambing, kg	4.4	3.6	0.216	0.043	-	0.567	-	0.127
Body weight at weaning, kg	14.4	12.6	0.764	0.369	-	0.731	-	0.253
Daily weight gain, g/day	222.2	200.0	16.23	0.391	0.018	0.474	0.949	0.488
Total weight gain from delivery, %	227.3	250.0	9.760	0.039	<0.001	0.069	0.967	0.488

Time: ANOVA effect for weeks of postpartum period intervals.

## Data Availability

The original contributions presented in this study are included in the article. Further inquiries can be directed to the corresponding author(s).
